# Identification and Analysis of Cation Channel Homologues in Human Pathogenic Fungi

**DOI:** 10.1371/journal.pone.0042404

**Published:** 2012-08-02

**Authors:** David L. Prole, Colin W. Taylor

**Affiliations:** Department of Pharmacology, University of Cambridge, Tennis Court Road, Cambridge, United Kingdom; University of Nebraska, United States of America

## Abstract

Fungi are major causes of human, animal and plant disease. Human fungal infections can be fatal, but there are limited options for therapy, and resistance to commonly used anti-fungal drugs is widespread. The genomes of many fungi have recently been sequenced, allowing identification of proteins that may become targets for novel therapies. We examined the genomes of human fungal pathogens for genes encoding homologues of cation channels, which are prominent drug targets. Many of the fungal genomes examined contain genes encoding homologues of potassium (K^+^), calcium (Ca^2+^) and transient receptor potential (Trp) channels, but not sodium (Na^+^) channels or ligand-gated channels. Some fungal genomes contain multiple genes encoding homologues of K^+^ and Trp channel subunits, and genes encoding novel homologues of voltage-gated K_v_ channel subunits are found in *Cryptococcus* spp. Only a single gene encoding a homologue of a plasma membrane Ca^2+^ channel was identified in the genome of each pathogenic fungus examined. These homologues are similar to the Cch1 Ca^2+^ channel of *Saccharomyces cerevisiae*. The genomes of *Aspergillus* spp. and *Cryptococcus* spp., but not those of *S. cerevisiae* or the other pathogenic fungi examined, also encode homologues of the mitochondrial Ca^2+^ uniporter (MCU). In contrast to humans, which express many K^+^, Ca^2+^ and Trp channels, the genomes of pathogenic fungi encode only very small numbers of K^+^, Ca^2+^ and Trp channel homologues. Furthermore, the sequences of fungal K^+^, Ca^2+^, Trp and MCU channels differ from those of human channels in regions that suggest differences in regulation and susceptibility to drugs.

## Introduction

Pathogenic fungi are widespread and cause a variety of diseases in humans, animals and plants, which are of huge medical and economic importance. In this study we focus on human fungal pathogens, which cause infections that are often difficult to treat and can be fatal [1], [2]. Fungal skin and nail infections such as tinea, which are caused most commonly by *Trichophyton* spp., affect more than twenty percent of the world’s population [3]. Various species of *Candida* are the most common cause of hospital-acquired fungal infections and cause opportunist infections in immunocompromised patients [1], [2], [4]. Airborne spores of *Aspergillus* spp. are widespread and these fungi cause disease via production of mycotoxins [5], induction of allergic reactions [6]–[8], or via localized and systemic infections [1], [2]. Systemic infections can also be caused by *Blastomyces dermatitidis, Coccidioides* spp. [9], [10] and *Paracoccidioides brasiliensis*; the latter affects more than 10 million people in South America [11]. Inhalation of airborne *Histoplasma capsulatum* is the most common cause of fungal respiratory infections [12], [13]. *Cryptococcus neoformans* and *Cryptococcus gattii* cause disease in around one million people each year, including immunocompetent individuals [14]–[16], and are estimated to cause more than 600,000 deaths [17]. The microsporidia *Encephalitozoon* spp. and *Enterocytozoon bieneusi* are an increasingly common cause of intestinal disease and diarrhoea in immunocompromised patients [18], [19]. Current therapies for many of the serious fungal diseases are inadequate or poorly tolerated, and resistance to therapeutic azole drugs is increasingly commonplace [20].

Ionic homeostasis within virtually all cells is maintained by an array of ion channels and transporters, which also allow rapid stimulus-evoked changes in cellular physiology. The diversity of cations (notably Na^+^, K^+^, H^+^ and Ca^2+^) with electrochemical gradients across biological membranes is much greater than for anions and there is a correspondingly diverse array of cation-selective channels [21]–[23]. Perturbing the activity of cation channels can profoundly affect cell function, and they are the targets of many clinically effective drugs [24]–[27]. This suggests that cation channels in fungal pathogens might play important roles in their physiology and may be targets for novel drugs.

Prominent cation channels include K^+^, Na^+^ and Ca^2+^ channels [22], the mitochondrial Ca^2+^ uniporter (MCU) [28], [29], many relatively non-selective channels such as Trp channels [22] and many ligand-gated channels [30]–[33]. The genome of the model fungal organism, *Saccharomyces cerevisiae*, encodes three homologues of mammalian cation channel subunits. These are the plasma membrane two-pore domain K^+^ (K_2P_) channel subunit TOK1 [34], [35]; the plasma membrane Ca^2+^ channel Cch1, which requires the additional Mid1 subunit for function [36], [37]; and the vacuolar membrane Trp channel subunit TrpY1 (also known as Yvc1) [38], [39]. The genome of *S. cerevisiae* does not encode homologues of MCU or Na^+^ channels, and also lacks genes encoding many other cation channel subunits (*see Results*). TOK1 homologues have been described in *Candida albicans* [40] and *Neurospora crassa* [41], [42], while genes encoding Ca^2+^ channels have recently been described in basal fungi [43], *Aspergillus* spp. [44] and *C. neoformans* [45]. In addition, purinergic P2X receptors, which are cation channels activated by adenosine triphosphate (ATP), have also been described in basal fungi [46]. However, there has been no systematic analysis of cation channels in many of the most important fungal pathogens.

Recent advances in genomics have resulted in whole-genome sequencing of many pathogenic fungi. In this study we examine these genomes comprehensively, using the sequences of diverse cation channel subunits from mammals, plants, fungi, bacteria and archaea, to search for genes that may encode cation channels. We identify genes likely to encode homologues of K^+^, Ca^2+^, Trp and MCU channels in many of the genomes examined. These genes are, however, less plentiful than in mammals and genes encoding homologues of many important mammalian cation channels, such as Na^+^ channels, are not present. Novel aspects of our findings include the identification of genes encoding previously undescribed homologues of K^+^, Ca^2+^ and Trp channel subunits in several pathogenic fungi; multiple homologues of Trp channel subunits in many fungi, including novel homologues more distantly related to TrpY1; novel homologues of voltage-gated K^+^ (K_v_) channel subunits in *Cryptococcus* spp. and some other fungi; and homologues of MCU in *Aspergillus* spp. and *Cryptococcus* spp.

## Results and Discussion

The genomes of most pathogenic fungi examined contain genes encoding homologues of K^+^, Ca^2+^ and Trp channel subunits, and some additionally have genes encoding homologues of MCU (**Table 1**). Many of these predicted proteins are not yet annotated as cation channels in fungal databases. In contrast, none of the fungal genomes examined contain genes encoding homologues of Na^+^ channels or the pore-forming subunits of many other cation channels, such as Orai1 (and its regulatory subunit, STIM1), purinergic receptors, cyclic nucleotide-gated (CNG) channels, hyperpolarization-activated cyclic nucleotide-sensitive non-selective (HCN) channels, N-methyl-D-aspartate (NMDA) receptors, nicotinic acetylcholine receptors, acid-sensing ion channels (ASICs), pannexins, two-pore Ca^2+^ (TPC) channels, mechanosensitive Piezo channels and voltage-gated Hv1 proton channels. It is also significant that genes encoding inositol 1,4,5-trisphosphate receptor (IP_3_R) and ryanodine receptor (RyR) subunits are absent from fungal genomes, despite the apparent importance of phospholipase C and IP_3_ in fungal physiology [47]–[49] and the ability of IP_3_ to elicit Ca^2+^ release from vacuolar vesicles of *S. cerevisiae* [50], *N. crassa* [51] and *C. albicans* [52]. The proteins responsible for the Ca^2+^-mobilizing effects of IP_3_ in fungi remain to be defined. No genes encoding homologues of any cation channel subunit were identified in the pathogenic microsporidia *Encephalitozoon intestinalis, Encephalitozoon cuniculi* and *E. bieneusi,* which have some of the smallest genomes known [53]. This is surprising given the importance of cation channels in most organisms. As *Encephalitozoon* spp. and *E. bieneusi* are obligate intracellular parasites, it may be that they do not require cation-selective channels to ensure ionic homeostasis, but rather rely on non-selective pathways that allow ionic continuity with the cytoplasm of the host cell. Other non-selective channels, ion transporters and exchangers are also likely to be present in fungi, which although beyond the scope of this study focussing on cation-selective channels, may also contribute substantially to cation fluxes and ion homeostasis.

**Table 1:** Cation channel homologues in pathogenic fungi.

Fungus	K^+^ channels	Ca^2+^ channels	Trp channels	MCU
*Saccharomyces cerevisiae*	TOK1 (NP_012442) (10) (K_2P_)	Cch1 (CAA97244) (24)	TrpY1 (NP_014730) (8)	NF (−)
*Trichophyton rubrum*	XP_003237995 (9) (K_2P_)	XP_003231641 (22)	XP_003238567 (8)XP_003239432 (8)	NF (+)
*Aspergillus clavatus*	XP_001268834 (9) (K_2P_) XP_001270765 (9) (K_2P_)	XP_001269155 (24)	XP_001271370 (8)XP_001268228 (8)	XP_001271905 (2) (+)
*Aspergillus flavus*	EED45164 (10) (K_2P_) EED53608 (9) (K_2P_)	EED50022 (24)	EED54784 (8) EED53521 (8)	EED55359 (2) (+)
*Aspergillus fumigatus*	XP_747058 (8) (K_2P_) XP_752795 (9) (K_2P_) XP_754857 (9) (K_2P_)	XP_752476 (24)	XP_001481630 (8)XP_751014 (8)	XP_751795 (2) (+)
*Coccidioides immitis*	NF	XP_001243065 (23)	XP_001246339 (8)XP_001240173 (8)	NF (+)
*Coccidioides posadasii*	NF	XP_003070141 (22)	XP_003066800 (8)XP_003069096 (8)	NF (+)
*Paracoccidioides brasiliensis*	XP_002791510 (12) (K_2P_)	XP_002794469 (22)	XP_002792043 (8)XP_002793104 (8)	NF (+)
*Candida albicans*	XP_712779 (9) (K_2P_)	XP_718390 (23)	XP_716049 (8)XP_717119 (9)	NF (−)
*Candida glabrata*	XP_448924 (9) (K_2P_)	XP_445066 (24)	XP_448082 (8)	NF (−)
*Candida tropicalis*	XP_002545324 (9) (K_2P_)	XP_002550113 (24)	XP_002547405 (8)XP_002547722 (7)	NF (−)
*Histoplasma capsulatum*	NF	HCEG_02563 (24)	HCEG_06995 (8)	NF (+)
*Blastomyces dermatitidis*	EGE81330 (8) (K_2P_)	EGE78212 (24)	EGE78766 (8)EGE79344 (9)	NF (+)
*Cryptococcus gattii*	XP_003191811(10) (K_2P_) XP_003192344 (6) (K_v_)	XP_003194030 (24)	XP_003191599 (8)	XP_003191929 (2) (+)
*Cryptococcus neoformans*	XP_568987 (10) (K_2P_) XP_569114 (6) (K_v_)	XP_570175 (24)	XP_566850 (8)	XP_566527 (2) (+)

Protein accession numbers are shown, except in the case of *H. capsulatum* for which transcript identifiers are shown (NCBI and Broad Institute of Harvard and MIT, *see Methods*). MCU denotes the human mitochondrial Ca^2+^ uniporter (NP_612366). Genes encoding homologues of MCU are also found in the genomes of: the Ascomycota *Aspergillus* spp., *Fusarium* spp., *Verticillium* spp., *Chaetomium globosum*, *Neurospora crassa*, *Magnaporthe grisea*, *Botrytis cinerea*, *Sclerotinia sclerotiorum*, *Stagonospora nodorum*, and *Pyrenophora tritici*-*repentis*; the Basidiomycota *Cryptococcus* spp., *C. cinerea* and *Ustilago maydis*; and the Chytridiomycota *A. macrogynus* and *Spizellomyces punctatus*. In contrast, genes encoding MCU homologues appear to be absent from the genomes of other fungi such as *E. cuniculi*, *E. intestinalis, E. bineusi, Saccharomyces* spp., *Schizosaccharomyces* spp., *Microsporum* spp., and other species of *Trichophyton*. Homologues of MICU1 (NP_006068), the Ca^2+^-sensing modulatory subunit of MCU, are also encoded by some fungal genomes, including (protein accession number or transcript identifier shown in parentheses): *T. rubrum* (XP_003233268), *A. clavatus* (XP_001273355), *A. flavus* (EED56817), *A. fumigatus* (XP_748987), *C. immitis* (XP_001245264), *C. posadasii* (XP_003071580), *P. brasiliensis* (XP_002792408), *H. capsulatum* (HCEG_05324.2), *B. dermatitidis* (EGE79123.1), *C. gattii* (XP_003192784) and *C. neoformans* (XP_569565), but appear to be absent from the other genomes examined. Homologues of the Cch1 auxiliary subunit Mid1 (NP_014108) in *S. cerevisiae* are also found in the following fungi: *T. rubrum* (XP_003235133.1), *A. clavatus* (XP_001273916), *A. flavus* (EED46777), *A. fumigatus* (XP_754048), *C. immitis* (XP_001242343), *C. posadasii* (XP_003069581), *P. brasiliensis* (XP_002790830), *C. albicans* (XP_710963), *C. glabrata* (XP_449502), *C. tropicalis* (XP_002551139), *H. capsulatum* (HCEG_04307.2), *B. dermatitidis* (BDDG_05843.1), *C. gattii* (XP_003192201) and *C. neoformans* (XP_569171). The number of predicted transmembrane domains in each protein is indicated in parentheses. For homologues of K^+^ channel subunits, the predicted family of K^+^ channel (K_2P_ or K_v_) is also indicated in parentheses. In addition to those shown, K_v_ channel subunit homologues were also identified in: the Basidiomycota *Coprinopsis cinerea* (XP_002910836), *Laccaria bicolour* (XP_001881176), *Serpula lacrymans* (EGN93868) and *Postia placenta* (EED81504); the Chytridiomycete *Allomyces macrogynus* (AMAG_10122.1, AMAG_16737.1, AMAG_16515.1, AMG_06554.1 and AMAG_15091.1); and the Zygomycete *Rhizopus oryzae* (RO3G_09031.3). The presence (+) or apparent absence (−) of homologues of MICU1 is indicated for each fungal genome, shown in parentheses after the MCU homologue annotation. NF denotes no homologues found.

### K^+^ Channels

**Figure 1:**
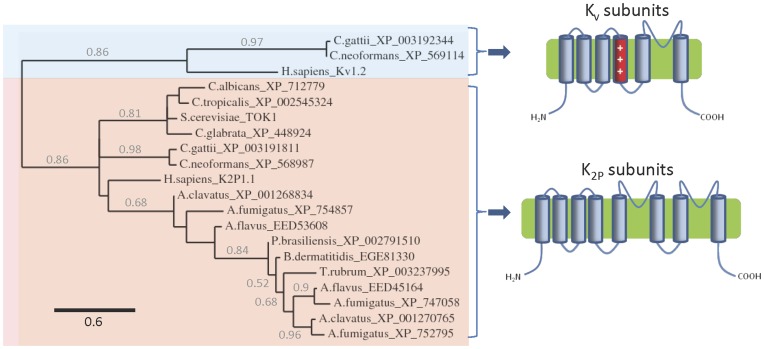
Fungal homologues of K^+^ channel subunits. Phylogram showing the relationship between the sequences of fungal and human K^+^ channel subunit sequences (*see Methods*: based on 44 high confidence positions from a multiple sequence alignment; gamma shape parameter 1.249; proportion of invariant sites zero). Branch length scale bar and branch support values >0.5 are shown. The predicted transmembrane topologies of the two distinct groups of putative K^+^ channel subunit (K_v_ and two-pore K_2P_ channel subunits) homologues are also shown.

**Figure 2:**
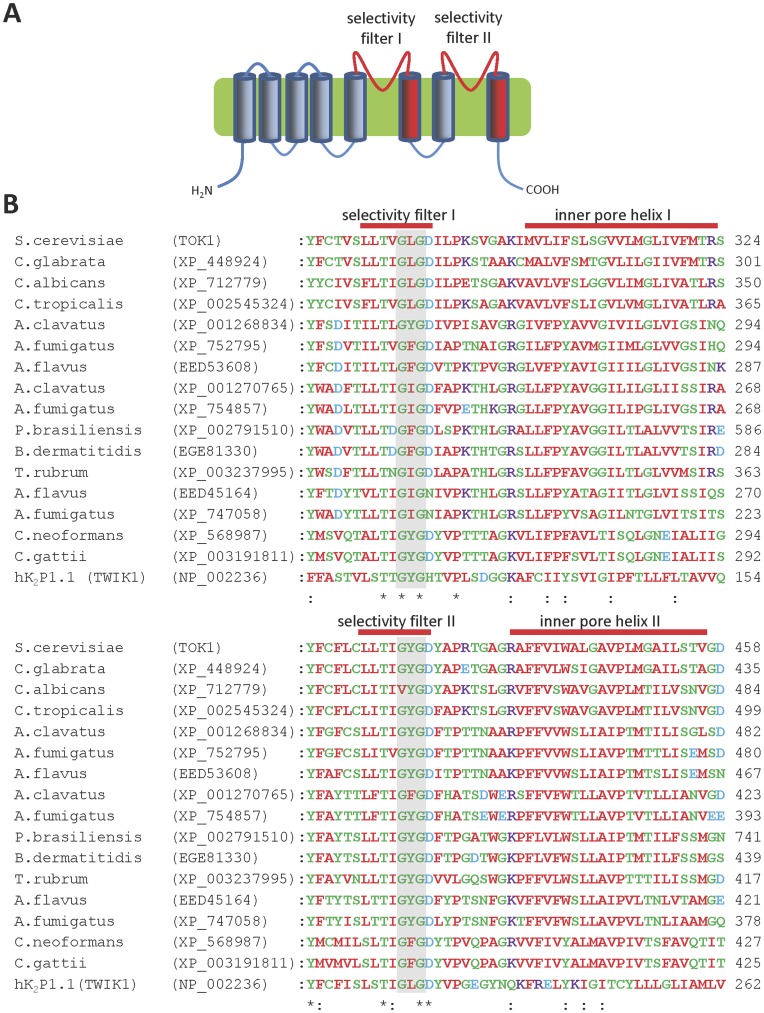
Fungal homologues of two-pore K^+^ (K_2P_) channel subunits. (**A**) Predicted transmembrane topology of fungal K_2P_ channel subunit homologues; (**B**) Multiple sequence alignment of the putative pore regions of fungal and human K_2P_ channel homologues. The shaded bar indicates the highly conserved GXG motif within the selectivity filter.

Genes encoding homologues of K^+^ channel subunits are found in the genomes of most pathogenic fungi examined, but are absent from *Coccidioides* spp. and *H. capsulatum* (**Table 1**). Most of these homologues are similar in predicted sequence and topological structure to the TOK1 channel subunit of *S. cerevisiae* (**Table 1**, **Figure 1** and **Figure 2**). Surprisingly, in addition to genes encoding homologues of two-pore K^+^ channels, the genomes of *C. neoformans* and *C. gattii* also contain genes encoding homologues of voltage-gated K_v_ channel subunits, which form a separate fungal K^+^ channel family (**Table 1** and **Figure 1**).

The putative two-pore K^+^ channel subunits contain a structure that is unique to fungal channels. Each subunit is predicted to contain eight transmembrane domains (TMDs), with two predicted selectivity filter regions, separated by two TMDs (**Figure 2A**) [34], [54], [55]. This predicted structure differs from the two-pore K^+^ channel subunits of other organisms, which have only four TMDs arranged like the last four TMDs of the larger fungal subunits [55]–[58]. Both types of two-pore channel are likely formed by dimerization of subunits [59], [60], which allows four pore-forming loops to create a central pore akin to that of mammalian K^+^ channels [56]. Multiple sequence alignments confirmed close sequence similarity of these proteins to the TOK1 two-pore K^+^ channel, and each contains the characteristic GXG selectivity filter motif of K^+^ channels within the putative pore regions (**Figure 2B**). Mutation of an aspartate residue immediately following the first GXG motif (D292N) dramatically alters the gating and K^+^ dependence of TOK1 [61]. Most TOK1 homologues have an aspartate residue at this locus, except for one homologue in *Aspergillus flavus* (EED45164) and another in *Aspergillus fumigatus* (XP_747058), which have an asparagine residue (**Figure 2B**). These homologues may therefore have substantially different gating properties to TOK1 and the other homologues. Another homologue which may have unique properties is that found in *C. albicans* (XP_712779), which has a VYG motif in place of a GXG motif in the second pore domain (**Figure 2B**). In contrast to the single gene encoding a two-pore K^+^ channel (TOK1) in *S. cerevisiae*, the genomes of *Aspergillus* spp. each contain two or three distinct genes (**Table 1**, **Figure 1** and **Figure 2B**). This suggests that K^+^ channels with different properties may be formed by these subunits, and also that heteromerization of subunits may increase the diversity of K^+^ channels in *Aspergillus* spp.

The physiological roles of TOK1 homologues are largely unknown, but in *S. cerevisiae* TOK1 plays a role in setting the plasma membrane potential [62], [63]. TOK1 channels are blocked by extracellular divalent cations [34], and their activity is decreased at acidic cytosolic pH [64], [65], enhanced by cytosolic ATP [65] and altered by changes in temperature [66]. Physiological modulators of mammalian two-pore K^+^ channels include fatty acids, voltage, post-translational modification and membrane stretch [57]. Whether these stimuli similarly modulate fungal homologues of TOK1 is unknown.

**Figure 3:**
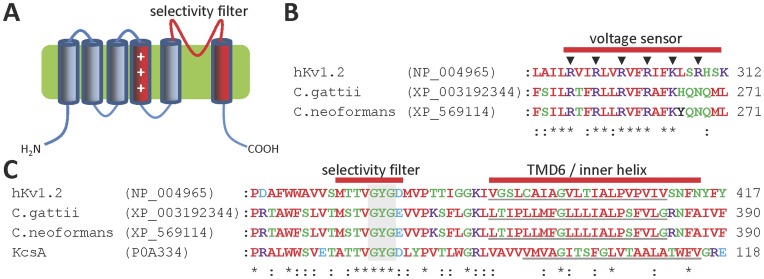
Fungal homologues of voltage-gated K^+^ (K_v_) channel subunits. (**A**) Predicted topology of fungal K_v_ channel subunit homologues; (**B**) Multiple sequence alignment of the putative voltage sensor TMD4 regions of human K_v_1.2 and fungal K_v_ channel homologues. Filled triangles above the alignment indicate the positions of conserved basic residues in K_v_1.2; (**C**) Multiple sequence alignment of the putative pore regions of human K_v_1.2 and fungal K_v_ channel homologues. Predicted pore-lining helices of each protein are underlined and the shaded bar indicates the highly conserved GXG motif within the selectivity filter.

The putative K_v_ channel subunits in *Cryptococcus* spp. are each predicted to have six TMDs, with TMD4 containing regularly spaced basic residues, similar to the voltage-sensing TMD4 domains of mammalian K_v_ channels [67], [68] (**Figures 3A and 3B**). They also have a single putative selectivity filter and pore-forming TMD6 region (**Figures 3A and 3C**). The fungal homologues have a conserved proline residue within TMD6, at a position equivalent to residue P405 of K_v_1.2 (**Figure 3C**). In K_v_ channels, a proline residue at this position introduces a kink in the pore-lining TMD6 α-helix, which facilitates gating in response to movement of the TMD4 voltage sensor [69]–[71]. This characteristically kinked TMD6 of K_v_ channels differs from many other K^+^ channels such as KcsA, which lack the proline residue and have straighter pore helices [72] (**Figure 3C**). Using sequences of the K_v_ channel homologues of *Cryptococcus* spp. as bait in further BLAST searches revealed that the genomes of only a few other fungi encode similar homologues of K_v_ channel subunits (**Table 1**). To the authors’ knowledge this is the first description of homologues of K_v_ channels in fungi.

The identification of genes encoding novel homologues of K_v_ channels in *Cryptococcus* spp. and several other fungi is surprising. These genes appear to be confined to the genomes of fungi within the phyla Basidiomycota, Zygomycota and Chytridiomycota, and appear to be entirely absent in Ascomycota. The K_v_ channel homologues contain putative voltage-sensing TMD4 domains and hence may be regulated by transmembrane voltage. Most K_v_ channels are activated by membrane depolarization [73], while a few are activated by hyperpolarization [74], [75]. Both types share sequence similarity in their voltage sensor domains [76], which makes it difficult to determine the polarity of their voltage-dependence on the basis of sequence alone. Experimental studies will be necessary to define the voltage sensitivity of these homologues. The majority of K_v_ channels are present and functional in the plasma membrane, where the greatest changes in transmembrane potential usually occur. It therefore seems likely that the fungal K_v_ channel homologues reside in the plasma membrane, although this will also require experimental analysis. The existence of putative K_v_ channel homologues in fungi suggests that dynamic changes in membrane potential may occur in fungi. The plasma membrane potentials of some fungi have been estimated. For example, the plasma membrane potential of *Pneumocystis jirovecii* has been estimated as −78 mV [77], that of *N. crassa* as −200 mV [78], [79] and those of various yeast cells as −50 to −120 mV [80]. Membrane potentials of some fungi are dependent on extracellular K^+^ concentration [81] and dynamic changes in membrane potential occur in the hyphae of *N. crassa* [82]. However, whether the membrane potentials of fungi change in response to environmental stimuli, and whether the K_v_ channel homologues identified here respond to such changes is unknown.

In many organisms K^+^ channels are found predominantly in the plasma membrane, but they are also present in the membranes of intracellular organelles such as mitochondria [83], endoplasmic reticulum (ER) [84], secretory vesicles [85], nuclei [86]–[89], endosomes [90] and vacuoles [91]. Physiological functions of K^+^ flux are similarly varied and include regulating membrane potentials, facilitating osmolyte homeostasis, modulating enzyme activity, initiating mitogenesis or apoptosis, and aiding transmembrane transport [56], [92]–[96]. Experimental studies will be required to determine the expression, cellular location and function of fungal K^+^ channels.

### Ca^2+^ Channels

**Figure 4:**
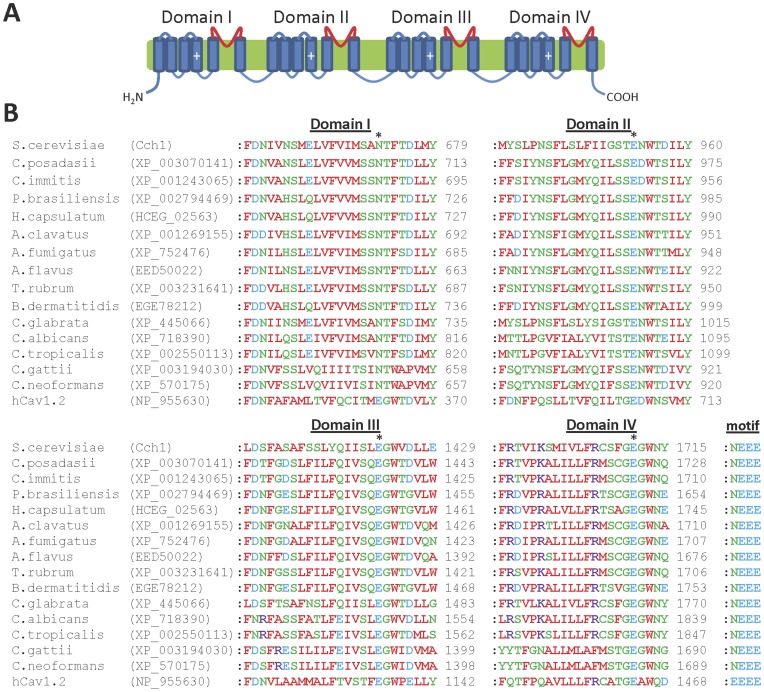
The pore regions of human Ca_v_ channels, Cch1 and fungal homologues are similar. (**A**) Predicted topology of Ca_v_ channels, with the pore loop regions of each domain highlighted in red; (**B**) Multiple sequence alignment of the putative pore loop regions from each domain of human Ca_v_1.2 and fungal Ca_v_ channel homologues. The overall motif present at the putative Ca^2+^ binding site locus (indicated by asterisks above the alignments) is indicated.

**Figure 5:**
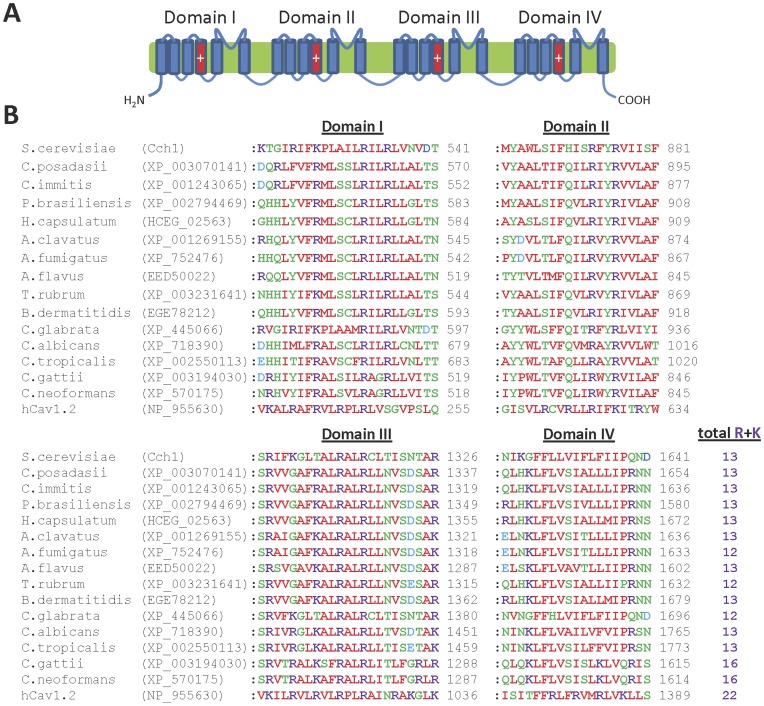
The voltage sensor regions of human Ca_v_ channels, Cch1 and fungal homologues are similar. (**A**) Predicted topology of Ca_v_ channels, with the voltage sensor TMD4 regions of each domain highlighted in red; (**B**) Multiple sequence alignment of the putative voltage sensor TMD4 regions from each domain of human Ca_v_1.2 and fungal Ca_v_ channel homologues. The total number of basic arginine and lysine residues present in all four putative voltage sensor TMD4 regions is indicated.

The genomes of all fungi examined (except the microsporidia) contain a single gene encoding a homologue of the plasma membrane Ca^2+^ channel Cch1 found in *S. cerevisiae* (**Table 1**), which is similar in sequence and topological structure to human voltage-gated Ca_v_ channels [36], [37] (**Figure 4A**). The same fungal genomes also have a gene encoding a homologue of Mid1, a regulatory subunit similar to the α2δ-subunits of mammalian Ca_v_ channels [97], which is necessary for the function of Cch1 [36], [37] (**Table 1**). In Ca_v_ channels, a Ca^2+^-binding site that contributes to ionic selectivity is formed by four acidic residues (EEEE), one from the selectivity filter region of each of the four domains (**Figures 4A and 4B**) [98], [99]. Each of the fungal homologues of Cch1 has a similarly placed acidic motif but with three, rather than four, acidic residues (**Figure 4B**). Sequences of the surrounding pore domains in human Ca_v_ channels and fungal homologues of Cch1 also differ substantially (**Figure 4B**). The fungal homologues have several regularly spaced basic residues in the TMD4 region of each domain (**Figure 5A and 5B**). This suggests that these regions may act as voltage sensors similar to those of mammalian Ca_v_ channels [99], although the latter have more basic residues (more than 20) than their fungal counterparts (typically 13, except *C. gattii* and *C. neoformans* which have 16) (**Figure 5B**) [99]. This suggests that fungal homologues of Cch1 may have a less pronounced voltage dependence compared to mammalian Ca_v_ channels, although this will require experimental analysis.

Plasma membrane Ca^2+^ channels are involved in many cellular processes. Ca^2+^ influx is a vital part of many physiological signalling pathways and it allows refilling of intracellular Ca^2+^ stores following release of intracellular Ca^2+^ [99]–[102]. The presence in all fungal genomes examined (except the microsporidia) of single genes encoding Cch1 and Mid1 homologues suggests a conserved function for Cch1/Mid1 Ca^2+^ channels, which are present in the plasma membrane in *S. cerevisiae* [36], [37]. Consistent with this, the fungal homologues of Cch1/Mid1 channels are involved in physiological processes such as mating [36], [37], [103], restoration of intracellular Ca^2+^ after release of Ca^2+^ from the ER [104], [105], growth, cell wall synthesis and virulence [106], [107], tolerance to cold stress and iron toxicity [108], high-affinity Ca^2+^ uptake during ionic stress [45], and hyphal growth [108]. Lack of Cch1 channels in *S. cerevisiae* impairs high-affinity Ca^2+^ uptake and leads to cell death in conditions of low Ca^2+^ concentration or when Ca^2+^ influx is required [36], [37]. The physiological regulators of Cch1/Mid1 channels are largely unknown, although charged TMD4 domains suggest possible regulation by voltage, and they are activated by mating pheromones [36], [37], [103] and by depletion of Ca^2+^ from the ER [104], [105].

### Mitochondrial Ca^2+^ Uniporters

**Figure 6:**
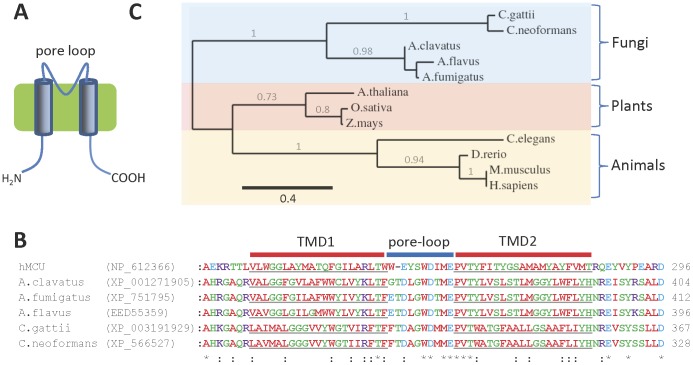
Homologues of MCU in pathogenic fungi. (**A**) Predicted topology of MCU channels, with the putative pore loop indicated; (**B**) Multiple sequence alignment of the TMDs and putative pore loop regions of human MCU and fungal homologues, with the predicted TMDs of each protein underlined; (**C**) Phylogram showing the relationship between the sequences of fungal, animal and plant MCU homologues (*see Methods*: based on 89 high confidence positions from a multiple sequence alignment; gamma shape parameter 2.969; proportion of invariant sites 0.04). Branch length scale bar and branch support values >0.5 are shown.

The genome of *S. cerevisiae* has been reported to lack genes encoding homologues of the recently described MCU, which provides a Ca^2+^ uptake pathway into mammalian mitochondria [28], [29]. This is consistent with a lack of effect of ruthenium red on mitochondrial Ca^2+^ uptake in *S. cerevisiae* [110]. In contrast, convincing homologues of MCU are encoded by the genomes of some pathogenic fungi (**Table 1**). As well as sequence similarity, the predicted topologies of fungal homologues are identical to MCU, with a single putative pore-loop region and the boundaries of the two predicted TMDs in identical positions (**Figures 6A and 6B**) [28], [29]. The sequences of MCU homologues in *Aspergillus* spp. and *Cryptococcus* spp. form a group that is phylogenetically distinct from plant and animal MCU homologues (**Figure 6C**). Like plant and human MCUs, most of the fungal homologues of MCU are predicted to contain cleavable N-terminal mitochondrial targeting sequences (MITOPROT; http://ihg.gsf.de/ihg/mitoprot.html) [111] (*data not shown*), suggesting that they may also be located in the inner mitochondrial membrane. Genes encoding homologues of MCU are present in pathogenic Ascomycetes (*Aspergillus clavatus*, *A. flavus* and *A. fumigatus*) and Basidiomycetes (*C. gattii* and *C. neoformans*) (**Table 1**). Genes encoding homologues of MCU are found in about 40% of all sequenced fungal genomes (*data not shown*). These include the genomes of various fungi in the Chytridiomycota, Basidiomycota and Ascomycota phyla (**Table 1**). Fungi that lack genes encoding homologues of MCU are also present in each phylum (**Table 1**). This absence of MCU homologues was in many cases confirmed in multiple, independently sequenced strains of fungi (*see Methods*), and by using the fungal homologues of MCU as bait in further BLAST searches. Those fungi that do have genes encoding homologues of MCU are closely related within their respective phyla (**Table 1**; [112], [113]. Together, these observations suggest that genes encoding homologues of MCU may have been lost on several independent occasions during the evolution of fungi.

Sequence similarity between fungal and mammalian homologues of MCU may identify residues that are functionally important. The loop between the two TMDs of MCU has been proposed to form the selectivity filter [28], [29]. This region contains a ^260^WDXMEPVT^267^ motif in human MCU that is conserved in the fungal homologues (**Figure 6B**). Further alignment of MCU homologues from such diverse organisms as plants, *Dictyostelium discoideum*, trypanosomes, *Monosiga brevicollis* and other fungi (*data not shown*) [28], [29], [114] shows that a core ^260^WDXXEP^265^ motif is most highly conserved (numbered for human MCU). Conserved acidic residues within the selectivity filter of Ca_v_ channels coordinate Ca^2+^ ions [99]. This suggests a possible role for the acidic residues, D261 and E264, of human MCU, and their equivalents in the fungal homologues, in the binding of Ca^2+^. Mutation of D261 or E264 in MCU compromises function, while the S259A mutant is functional but resistant to the inhibitor, Ru360 [28]. Fungal homologues of MCU differ from human MCU at the position equivalent to residue 259 (they have leucine or alanine in place of serine), suggesting that they may have different pharmacological profiles.

We also searched the genomes of pathogenic fungi for genes encoding homologues of MICU1, a protein containing EF-hands that may form an auxiliary Ca^2+^-sensing subunit that modulates MCU activity [115]. Expression of MICU1 and MCU is highly correlated in many organisms and tissues [28], [29]. Indeed, this correlation was central to the comparative genomics approach that led to the molecular identification of MCU [28], [29]. We found that like genes encoding homologues of MCU, genes encoding homologues of MICU1 are present in *Aspergillus* spp. and *Cryptococcus* spp. but appear to be absent in *Candida* spp. and *S. cerevisiae* (**Table 1**). This further suggests that a MCU-MICU1 Ca^2+^ uptake pathway is present in some pathogenic fungi but not in others, and as reported previously [28], [29] it is absent in *S. cerevisiae*. It is intriguing that genes encoding homologues of MICU1, but not MCU, are present in some fungi (**Table 1**). It is unclear what role homologues of MICU1 might play in these fungi, which include *T. rubrum, Coccidioides* spp., *P. brasiliensis*, *H. capsulatum* and *B. dermatitidis* (**Table 1**). Mammalian MCU plays a role in processes such as metabolism, apoptosis and cell signalling [114]. The physiological implications of MCU channels and MICU1 in pathogenic fungi remain to be explored.

### Trp Channels

**Figure 7:**
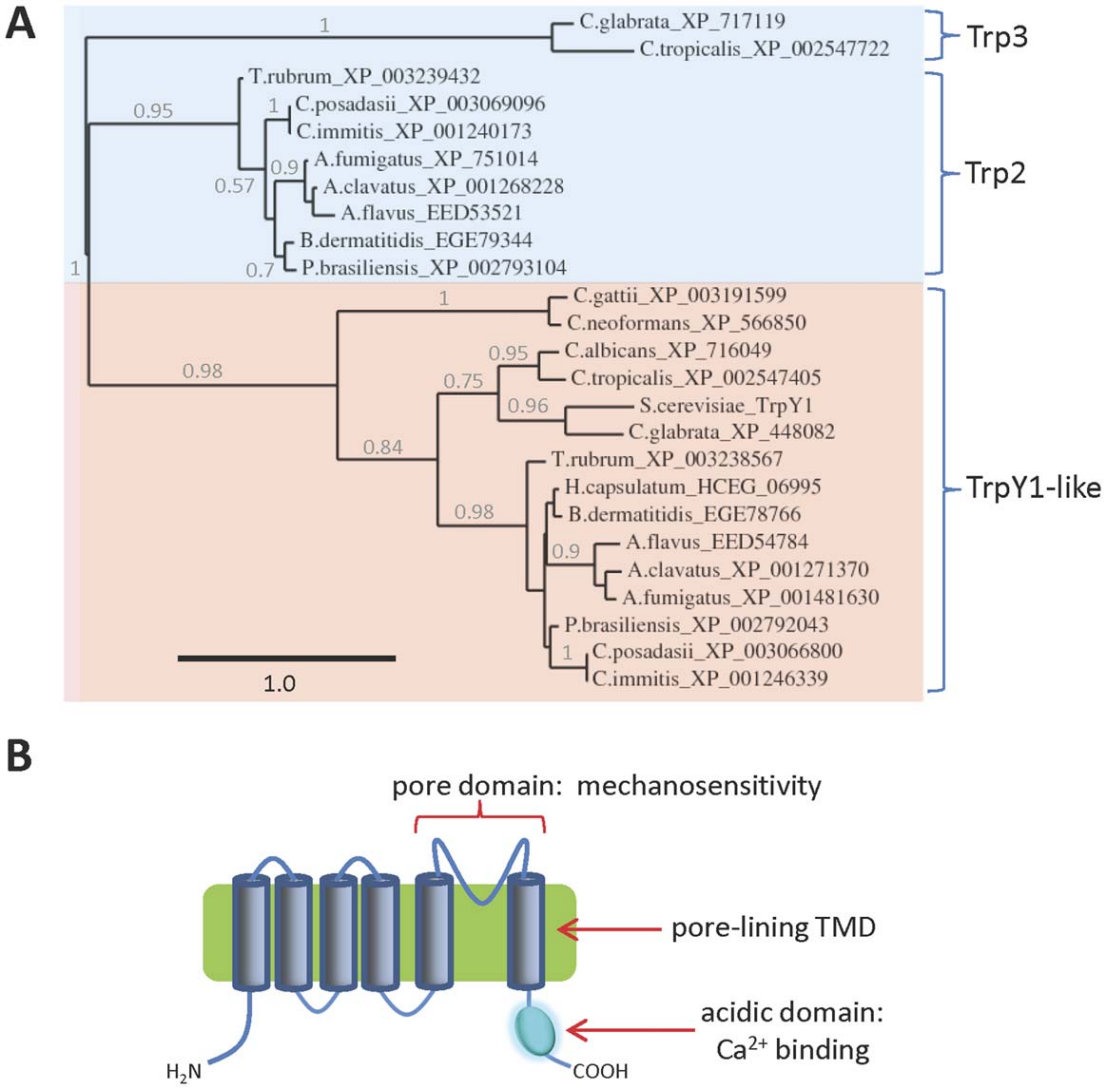
Fungal homologues of Trp channel subunits. (**A**) Phylogram showing the relationship between the sequences of fungal Trp channel subunit homologues (*see Methods*: based on 176 high confidence positions from a multiple sequence alignment; gamma shape parameter 1.209; proportion of invariant sites zero). The three distinct groups of Trp channel subunit homologue are shown. Branch length scale bar and branch support values >0.5 are shown; (**B**) Predicted topology of Trp channel subunits. The putative pore region responsible for mechanosensitivity, as well as the C-terminal acidic domain involved in Ca^2+^ sensitivity are indicated.

Genes encoding homologues of Trp channel subunits are found in all fungal genomes examined, except those of the microsporidia (**Table 1**). Some species, including *S. cerevisiae*, *Cryptococcus* spp. and *H. capsulatum*, have a single gene (**Table 1**, **Figure 7A**), but others have two genes (*T. rubrum*, *Aspergillus* spp., *Coccidioides* spp., *Paracoccidioides* spp., *Candida* spp. and *B. dermatitidis*) (**Table 1** and **Figure 7A**). Fungal homologues of Trp channel subunits form at least three distinct groups, here termed TrpY1-like (the largest group), Trp2 and Trp3 (**Figure 7A**). The fungal homologues have at least six predicted TMDs, suggesting that their topologies are similar to TrpY1 and human Trp channel subunits (**Figure 7B**) [22], [116].

**Figure 8:**
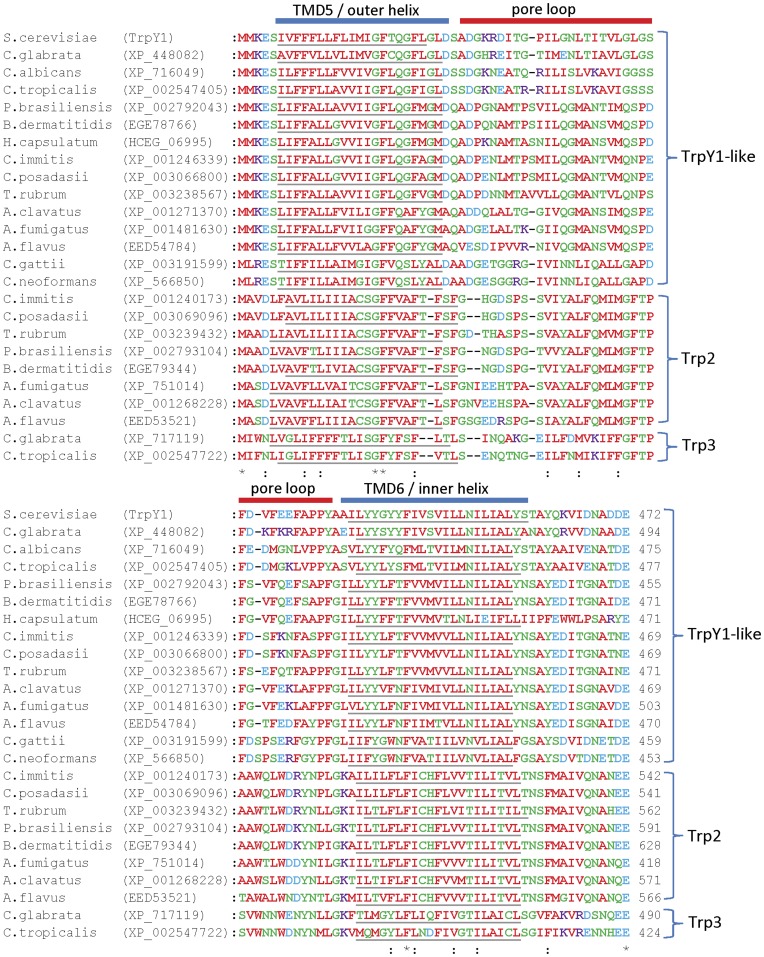
Fungal homologues of Trp channel subunits show similarity to the pore region of TrpY1 involved in mechanosensitivity. Multiple sequence alignment of the putative pore-domain TMDs and pore loop regions of fungal Trp channel subunit homologues, with the predicted TMDs of each protein underlined. The three distinct groups of Trp channel subunit homologue are indicated.

**Figure 9:**
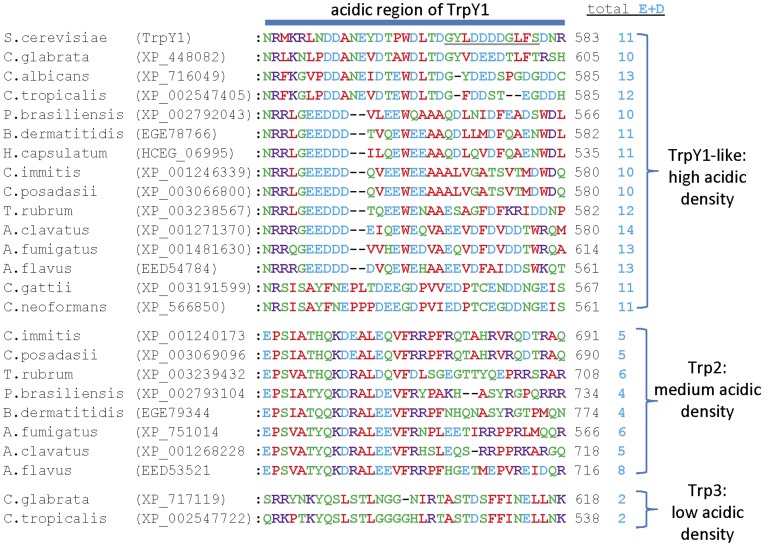
Fungal homologues of Trp channel subunits show similarity to the C-terminal acidic region of TrpY1 involved in Ca^2+^ sensitivity. Multiple sequence alignment of the C-terminal acidic region of TrpY1 involved in Ca^2+^ sensitivity with fungal Trp channel homologues. A region of TrpY1 critical for Ca^2+^ sensitivity [117] is shown underlined. The total number of acidic residues present in this region for each homologue is indicated. Also indicated are the three distinct groups of Trp channel subunit homologues.

In *S. cerevisiae*, Ca^2+^ release from vacuolar stores occurs via TrpY1 channels that are activated by membrane stretch [117], Ca^2+^ [117] and phosphatidylinositol 3,5-bisphosphate (PI(3,5)P_2_) [118]. Activation by membrane stretch is likely mediated by the pore-forming domain [119] (**Figure 7B**), while activation by Ca^2+^ is dependent on a C-terminal region containing many acidic residues that may form a Ca^2+^-binding site [117] (**Figure 7B**). The sequences of the pore-forming regions divide the fungal homologues into their three major families (**Figure 8**). Sequence similarity between TrpY1 and the TrpY1-like homologues is pronounced in this region (**Figure 8**), suggesting that pore-mediated mechanosensitivity [119] may be a conserved feature of these channels. Most notable among the conserved residues are a glycine-phenylalanine motif in the middle of TMD5 (^393^GF^394^ in TrpY1), a phenylalanine in TMD6 (^444^F in TrpY1), and an acidic residue or motif following TMD6 (^471^DE^472^ in TrpY1) (**Figure 8**). These conserved residues in the pore domain of fungal Trp channels may play important roles in channel gating or conductance, although this will require experimental investigation. Many fungal homologues of Trp channel subunits contain highly acidic regions in their C-terminal domains (**Figure 9**), which are similar to the acidic region involved in activation of TrpY1 by Ca^2+^ [117]. The density of acidic residues is greatest for the TrpY1-like homologues (**Figure 9**) suggesting that they, like TrpY1, may be regulated by cytosolic Ca^2+^. There are fewer acidic residues in the Trp2 homologues and very few in the Trp3 homologues (**Figure 9**). Experimental studies will be required to assess the possibility that these regions confer differential Ca^2+^ regulation on fungal homologues of Trp channels. The regions of TrpY1 responsible for activation by PI(3,5)P_2_ have not been determined. Basic residues within the N-terminal region of mammalian TrpML channels are involved in activation by PI(3,5)P_2_ [118], but these residues are not conserved in either TrpY1 or the other fungal homologues of Trp channel subunits (*data not shown*). From these comparisons of sequences with known determinants of TrpY1 regulation, we suggest that the TrpY1-like group of homologues may form mechanosensitive and Ca^2+^-modulated channels. The physiological regulators of the Trp2 and Trp3 groups of homologues are more difficult to predict.

Mammalian Trp channels play diverse roles both in release of Ca^2+^ and other ions from intracellular stores [23], [120]–[122], and in the influx of Ca^2+^ across the plasma membrane [116], [123], [124]. It is therefore interesting that genes encoding three distinct groups of Trp homologues are present in the genomes of several pathogenic fungi. One of these groups shares a high degree of sequence similarity with the vacuolar TrpY1 channel of *S. cerevisiae*, while the others are more distantly related. Further experimental work will be required to assess whether fungal Trp channel homologues form channels permeable to Ca^2+^ or other ions within the membranes of intracellular organelles such as vacuoles, ER or Golgi, or within the plasma membrane, and to define their physiological roles and regulation.

### Relevance to Therapy

Currently used antifungal drugs include azoles, allylamines and the macrolides amphotericin and nystatin, all of which are thought to act mainly via effects on ergosterol [125], [126]. Other drugs include pyrimidine analogues which affect protein synthesis, and sulphonamides. These drugs often have limited efficacy together with substantial side-effects, and emergence of drug resistance is an increasing problem [20]. New drugs to treat fungal infections are therefore needed. In many organisms K^+^, Ca^2+^ and Trp channels are essential components of cellular signalling and homeostatic pathways, and they are drug targets in humans [24], [27]. While the human genome contains genes encoding at least 78 K^+^ channel subunits, 11 Ca_v_ channel α-subunits and more than 30 Trp channel subunits [22], the genomes of pathogenic fungi each contain only very small numbers of genes encoding homologues of cation channel subunits (**Table 1**). This striking lack of redundancy amongst cation channels in pathogenic fungi suggests that they might be effective therapeutic targets. Furthermore, some anti-fungal drugs affect K^+^, Ca^2+^ or Trp channel function. For example, azole drugs such as clotrimazole inhibit Trp channels [127], [128], K^+^ channels [129], [130] and Ca^2+^ channels [131].

Although they have sequence motifs similar to mammalian K^+^ channels, and two pore domains similar to human two-pore K^+^ (K_2P_) channel subunits, the fungal homologues of TOK1 have a topology and putative structure that is unique to fungi. They are also likely to have a unique gating mechanism [132]. These factors suggest that they may be attractive pharmacological targets. This suggestion gains some support from evidence that a viral toxin that activates TOK1 in *S. cerevisiae* causes cell death, due to excessive K^+^ flux [133], and a TOK1 homologue in *C. albicans* increases sensitivity to human salivary histatin-5 [40]. Activators or inhibitors of TOK1 homologues may therefore be novel anti-fungals.

A diverse range of agents affecting Ca^2+^ channels or Ca^2+^ signalling pathways are also toxic to fungi [134]–[138], and Ca^2+^ channels are involved in the survival of fungal cells after azole-induced stress [97], [138]. The differing pore sequences of human Ca_v_ channels and fungal homologues of Cch1 (**Figure 4B**) suggest that analogues of Ca_v_ channel modulators, which often bind within the pore region [139]–[143], may exhibit selectivity for fungal Cch1 homologues over Ca_v_ channels. Mitochondrial Ca^2+^ uptake may be involved in the anti-fungal effects of some peptides [144] and mitochondrial function is linked to drug sensitivity in several fungi [145]–[147], suggesting that fungal homologues of MCU may be attractive novel targets for anti-fungal drugs. Ru360 is a potent inhibitor of MCU [148], and analogues of this drug might possess selective anti-fungal properties against those fungi that contain genes encoding MCU homologues, such as *Aspergillus* spp. and *Cryptococcus* spp. Pharmacological modulators of Trp channel function, which are increasingly being developed as potential therapeutic drugs against human targets [27], may also show anti-fungal activity via effects on fungal homologues of Trp channels. Indole and other aromatic compounds such as quinoline and parabens activate TrpY1 [149] and may potentially have anti-fungal activity.

This study presents the opportunity for cloning and functional characterization of cation channels in pathogenic fungi, and suggests that rational design of drugs targeted against these channels may be an effective route to new therapies.

## Materials and Methods

### Genomes Analyzed

The genomes of the following pathogenic fungi were examined (NCBI and the Broad Institute of Harvard and MIT [150], February 2012): the Ascomycota Trichophyton rubrum CBS 118892, Aspergillus clavatus NRRL 1, Aspergillus flavus NRRL3357, Aspergillus fumigatus Af293 [151], Candida albicans SC5314 [152], Candida glabrata CBS 138, Candida tropicalis MYA-3404, Coccidioides immitis RS, Coccidioides posadasii C735 delta SOWgp, Paracoccidioides brasiliensis Pb01, Blastomyces dermatitidis ATCC 18188 and Histoplasma capsulatum H88; the Basidiomycota Cryptococcus gattii WM276 and Cryptococcus neoformans JEC21; and the Microsporidia Encephalitozoon intestinalis ATCC 50506, Encephalitozoon cuniculi GB-M1 and Enterocytozoon bineusi H348 [153]. The genome of S. cerevisiae S228c was also used. To corroborate the absence of genes encoding particular channel homologues, the genomes of additional strains were analyzed, including: S. cerevisiae CAT-1, A. fumigatus A1163, C. posadasii str. Silveira, P. brasiliensis Pb03, P. brasiliensis Pb18, C. albicans WO-1, H. capsulatum NAm1, B. dermatitidis ER-3, and C. neoformans var. neoformans B-3501A.

### BLAST Searches, Alignments and Topology Analysis

Analysis of genomes, sequence alignments and topology analysis were conducted as reported previously [58], [154]. BLASTP and TBLASTN analyses to identify homologues of Ca^2+^, Na^+^ and non-selective cation channel subunits were carried out using the following human sequences (protein accession number in parentheses): full-length or pore sequences of IP_3_R1 (Q14643.2; pore region residues 2536–2608) or RyR1 (P21817.3; pore region residues 4877–4948), and sequences of human TrpA1 (NP_015628; N-truncated sequence residues 765-end), TrpV1 (NP_061197; N-truncated sequence residues 430-end), TrpC1 (P48995; N-truncated sequence residues 350-end), CNGA1 (EAW93049; transmembrane sequence residues 200–420), CNGB1 (NP_001288), HCN2 (NP_001185.3; full-length, and TMD residues 200–470), NMDA receptor NR1 (Q05586), NMDA receptor N2 (Q12879), AMPA receptor GRIA1 (P42261.2), kainate receptor GRIK1 (P39086), nAChR-alpha1 (ABR09427), purinergic receptor P2X4 (NP_002551.2), pannexin-1 (AAH16931), Orai1 (NP_116179.2), STIM1 (AAH21300), TPC1 (NP_001137291.1), TPC2 (NP_620714.2), TrpP1 (NP_001009944), TrpP2 (NP_000288), TrpM1 (NP_002411), TrpML1 (NP_065394), CatSper1 (Q8NEC5.3), acid-sensing ion channel-1 (ASIC1) (P78348.3), mitochondrial uniporter (NP_612366.1), Ca_v_1.2 (NP_955630.2), Na_v_1.1 (NP_001189364), Piezo-1 (NP_001136336), Piezo-2 (NP_071351) and NALCN (AAH64343). Sequences of the *S. cerevisiae* Ca^2+^ channel Cch1 (CAA97244), Mid1 (NP_014108) and TrpY1 (NP_014730), as well as *Arabidopsis thaliana* TPC1 (AAK39554) were also used to search for fungal homologues. The sequence of the MCU auxiliary subunit MICU1 (NP_006068.2) was also used. Searches to identify K^+^ channel homologues were carried out using the following sequences of diverse human K^+^ channels (protein accession number in parentheses): K_v_1.2 (NP_004965.1), K_v_7.1 (NP_000209.2) and K_v_11.1 (hERG1) (Q12809.1); K_ir_1.1 (ROMK1) (NP_000211.1), K_ir_2.1 (IRK1) (NP_000882.1), K_ir_3.1 (GIRK1) (NP_002230.1), K_ir_4.1 (P78508.1), K_ir_5.1 (Q9NPI9.1), K_ir_6.1 (K_ATP_1) (Q15842.1), K_ir_6.2 (NP_000516.3) and K_ir_7.1 (CAA06878.1); K_2P_1.1 (TWIK1) (NP_002236.1), K_2P_2.1 (TREK1) (NP_001017425.2), K_2P_3.1 (TASK1) (NP_002237.1), K_2P_13.1 (THIK1) (NP_071337.2), K_2P_16.1 (TALK1) (NP_001128577.1) and K_2P_18.1 (TRESK2) (NP_862823.1); K_Ca_1.1 (BK) (NP_001154824.1), K_Ca_2.1 (SK1) (NP_002239.2), K_Ca_2.2 (SK2) (NP_067627), K_Ca_3.1 (IK/SK4) (NP_002241.1) and K_Ca_4.1 (SLACK/K_Na_) (NP_065873.2). Other K^+^ channel sequences were also used to search for fungal homologues, including: bacterial KcsA (P0A334), bacterial cyclic nucleotide-gated MlotiK1 (Q98GN8.1), archaeal depolarization-activated K_v_AP (Q9YDF8.1), archaeal hyperpolarization-activated MVP (Q57603.1), archaeal Ca^2+^-activated MthK (O27564.1), and TOK1 from *S. cerevisiae* (CAA89386.1). Plant K^+^ channel sequences were also used, including: the vacuolar outwardly rectifying, Ca^2+^-regulated vacuolar two-pore TPK1 channel (NP_200374.1); vacuolar KCO3 (NP_001190480.1); the pollen plasma membrane TPK4 (NP_171752.1), the inward rectifier KAT1 (NP_199436.1), the outward rectifier SKOR (pore region of NP_186934.1, residues 271–340 to avoid ankyrin hits), and AKT1 (NP_180233.1). We also searched for homologues of Hv1 proton channel subunits (NP_115745.2). Default BLAST parameters for assessing statistical significance and for filtering were used (*ie*. an Expect threshold of 10, and SEG filtering).

Several procedures ensured that hits were likely homologues of cation channel subunits. Firstly, the presence of multiple transmembrane domains was confirmed using TOPCONS [155]. Secondly, reciprocal BLASTP searches (non-redundant protein database at NCBI) were made, using the identified fungal hits as bait, and only proteins that gave the original mammalian protein family as hits were analyzed further. Thirdly, the presence of conserved domains was confirmed using the Conserved Domains Database (NCBI). In addition, for homologues of K^+^ channel subunits, only hits with regions of sequence similarity that encompassed the selectivity filter sequence of the K^+^ channel subunit used as bait were acknowledged. Also, where possible, pore homology was confirmed by sequence alignment using ClustalW2.1 (European Bioinformatics Institute). Multiple sequence alignments were made using ClustalW2.1 and physiochemical residue colours are shown. Where shown, asterisks below the alignment indicate positions that have a single fully conserved residue, while colons indicate positions that have residues with highly similar properties (scoring >0.5 in the Gonnet PAM 250 matrix, ClustalW2). For phylogenetic analysis, multiple sequence alignments were made with MUSCLE v3.7 using default parameters. After using GBLOCKS at high stringency to remove regions of low confidence, and removing gaps, Maximum Likelihood analysis was carried out using PhyML v3.0 (WAG substitution model; 4 substitution rate categories; default estimated gamma distribution parameters; default estimated proportions of invariable sites; 100 bootstrapped data sets). Phylogenetic trees were depicted using TreeDyn (v198.3). MUSCLE, GBLOCKS, PhyML and TreeDyn were all functions of Phylogeny.fr (http://www.phylogeny.fr/) [156].
